# Overview on Patient Centricity in Cancer Care

**DOI:** 10.3389/fphar.2017.00698

**Published:** 2017-10-05

**Authors:** Šarunas Narbutas, Kristina York, Barry D. Stein, Kara Magsanoc-Alikpala, Yoshiyuki Majima, Zoltan Kalo, Timea Almasi, Andras Inotai

**Affiliations:** ^1^Lithuanian Cancer Patient Coalition (POLA), Kaunas, Lithuania; ^2^Faculty of Law, Vilnius University, Vilnius, Lithuania; ^3^Independent Researcher, Innsbruck, Austria; ^4^Colorectal Cancer Association of Canada, Montreal, QC, Canada; ^5^ICanServe Foundation, Pasig, Philippines; ^6^Pancreatic Cancer Action Network Japan, Chiyodaku, Tokyo, Japan; ^7^Syreon Research Institute, Budapest, Hungary; ^8^Faculty of Social Sciences, Institute of Economics, Eötvös Loránd University, Budapest, Hungary

**Keywords:** patient preference, patient empowerment, oncology, guideline, patient organization, cancer care

## Abstract

Successful implementation of treatment in cancer care partially depends on how patients' perspectives are taken into account, as preferences of health care professionals and patients may differ. Objectives of this exploratory research were (I) to identify patient preferences and values (PPVs) in cancer care as indicated by patient organizations (POs), (II) to determine how these PPVs are captured in cancer care guidelines and (III) to review how guidelines take into account these PPVs. Based on a survey developed and completed by 19 POs, a literature review was conducted to analyse how patient perspectives are incorporated in oncology treatment guidelines. Based on survey results traditional health technology assessment value propositions of oncology care, such as extended life, treatment-free remission and pain reduction, were also highly rated by POs. However, the heterogeneity of cancer PPVs were clearly reflected in the survey results. PPVs in cancer care guidelines were mostly limited to those micro-level aspects that are strictly related to health care provision, such as side-effects and comorbidities. Patient experience, emotional support and convenience of care were relatively neglected fields in the reviewed guidelines. Patient engagement was rarely presented in the guideline development phase. POs believe that patients should be encouraged to take an active role in their own care due to the heterogeneity of cancer patients and PPVs. Even if patient-centricity is a leading paradigm in cancer policy, based on our research it is not yet standard practice to include patients or POs at all appropriate levels of decision-making processes that are related to their health and well-being. Patient engagement should be an integral part of cancer care decision-making. This complexity must be reflected throughout policy making, avoiding a population level “one-size-fits-all” solution.

## Introduction

Representing a significant economic burden on health care systems worldwide, cancer is associated with a high level of morbidity and mortality in virtually every country (Jonsson et al., 2016). Prevalence is increasing along with incidence (Siegel et al., 2015). In 2012, there were 14 million cases of diagnosed cancer patients, while the number of cancer-related deaths was estimated to be 8.2 million (McGuire, 2016). The global cancer mortality increased by 17% between 2005 and 2015 (Wang et al., 2016). During the same period the global burden of neoplasms measured in Disability Adjusted Life Years (DALYs) increased by 11.6% (Kassebaum et al., 2016). The global cancer burden will further increase in the near future, the number of new cases is projected to achieve 22.2 million by 2030 (Vineis and Wild, 2014). Societies with established health care systems dedicate significant resources to providing access to cancer diagnostics and therapies, resulting in improved life expectancy for many oncological conditions (Jonsson et al., 2016). However, extension of life implies extended use of therapies and other health care services, both being accompanied by growing patient needs (IOM, 2001; van Baal et al., 2008). There still is a large variation among nations when it comes to cancer care and nationally allocated expenditures (Luengo-Fernandez et al., 2013; Kimman et al., 2015).

Value of cancer care treatments in terms of benefits of increased survival and/or increased quality of life poses a challenge especially in lower income countries, while simultaneously considering their affordability. The evaluation of the benefits derived from cancer therapies is difficult as the extent of the benefit may be highly dependent on the components of the measurement and the individual patient perspective, i.e., a therapy can be valued differently by patients, caregivers, payers, and/or society (Basch, 2016). Nonetheless, assessing health gains should not be done without asking the beneficiaries (i.e., patients) about their needs and expectations of care they receive (Tremblay et al., 2015; King et al., 2016). Nevertheless, heterogeneity of patient preferences and values (PPVs) poses a complex problem, and although seeking a simple population-based solution may seem attractive from a policy perspective, it may not bring the needed tangible real-life benefits.

Multiple value frameworks have been recently published with the purpose of combining patients', providers', and payers' priorities, including those developed by the American Society of Clinical Oncology (ASCO) (Schnipper et al., 2015, 2016), the European Society for Medical Oncology (ESMO) (Cherny et al., 2015), the Institute for Clinical and Economic Review (ICER) (ICER, 2017), the Memorial Sloan Kettering Cancer Center (MSKCC) (MSKCC, 2017), the National Comprehensive Cancer Network (NCCN) (NCCN, 2017) and the National Cancer Institute—U.S. Department of Health and Human Services (Epstein and Street, 2011). As stated by Basch (2016), the primary focus of several guidelines is setting up a benchmark for institutional standards by taking into account the clinical evidence and the associated drug costs in order to facilitate treatment choices for payers, providers and patients (Basch, 2016). Nonetheless, these frameworks do not appear to fully address the complex issue of patient heterogeneity and patient preferences.

Beyond direct health benefits there are other aspects with potential added value to cancer therapies. Molecular diagnostics supported by genomic profiling allow applied novel targeted therapies to effectively treat patients, tailoring the treatment to target their unique tumor characteristics (Al-Rohil et al., 2016). Several patient surveys, including Patient Reported Experience Measures (PREMs), have been developed to identify priorities of patient experiences and preferences (Weldring and Smith, 2013; Taylor et al., 2015; Tremblay et al., 2015; Windham et al., 2015; Burns et al., 2016; Halpern et al., 2016). Alongside this gradual shift toward personalized medicine, importance of patient engagement is also increasingly recognized (Tremblay et al., 2015; King et al., 2016; Schnipper et al., 2016). One driver behind their increased involvement is the recently initiated patient network for “big data” collection on individuals' genetic and therapeutic characteristics (CMBRP, 2016). However, patient representatives often argue that current value frameworks and guidelines have limitations in reflecting or even admitting the importance of patients' preferences (Pitts and Goldberg, 2015).

The objective of this research was (I) to identify PPVs in cancer care and treatment as indicated by POs surveyed on behalf of their patient population; (II) to determine how these PPVs are captured in the guidelines on both a micro and macro level and finally (III) to review how patient representation in clinical and policy decisions are facilitated by the guidelines. Authors are not aware of studies on PPVs from wide array of Patient Organizations (POs) from countries on different continents.

## Methods

An exploratory research was conducted to identify key terms that can be considered as relevant value propositions for patients undergoing cancer treatment based on the review of selected publications (Epstein and Street, 2007, 2011; Pitts and Goldberg, 2015; Schnipper et al., 2015, 2016; Windham et al., 2015). Lead representatives of participant POs selected these papers partly because they had been widely discussed in global conferences and they were recommended by clinical experts. After selection of patient relevant value propositions an electronic survey was developed and completed by leaders of 19 POs from four continents to explore relative importance of these PPVs. Nine of the POs initiated this research project via the Global Action for Cancer Patients platform, and an additional 10 POs—all of them are national leaders in their own respective disease areas—joined the survey. Finally, a systematic literature review—using Pubmed as a research engine—was conducted to evaluate how clinical cancer care guidelines incorporate the identified PPVs.

### PO survey

The survey filled in by PO leaders intended to explore what patients deem to be of most value to them. The final survey included three groups of items/topics: The first set of questions were focusing on descriptive general characteristics of the POs (way of funding, number of payed and voluntary staff). The second group of items were related to POs perception on PPVs (aspects of care and their importance rated on a 5-point ordinal scale, fields for improvement in cancer care formulated as an open-ended question), while the third group contained open-ended questions on involvement of POs into decision making [involvement of patient representatives to health technology assessment (HTA)]. (For the actual questions, please refer to the headlines of the corresponding tables) POs were informed about the objectives of the survey, including the notion of presenting aggregated results in a scientific manuscript. As the survey was filled in by POs and only general policy questions were surveyed without collecting any clinical personal data, after legal consuelling no ethical approval was considered to be necessary for the study.

### Development of data extraction spreadsheet

Outcomes of the explotarory research and survey results were used to develop a hierarchical classification and data extraction spreadsheet to collect information on PPVs in the clinical cancer care guidelines. The data extraction focused on the presence of three main categories in guidelines, including: (I) patient empowerment related PPVs, (II) health outcomes related PPVs, and (III) patient management related PPVs in the guidelines (see Table 1). The draft data collection spreadsheet was pre-tested using literature references in the initial exploratory research and appeared to be a relevant tool for further data collection.

**Table 1:** Categories to be registered during full text review.

**1. Patient empowerment related PPVs in the guidelines**
1.1. Patient perspective is present in the guideline
1.2. Patient heterogeneity
1.3. Patient voice in treatment choice
1.4. Patient support group/organization
1.5. Quality improvement based on patients' feedback
**2. Health outcomes related PPVs in the guidelines**
2.1. Side-effect management
2.2. Emotional support
2.3. Palliative care
2.4. Comorbidities
2.5. Extended life
2.6. Treatment-free remission
**3. Patient management related PPVs in the guidelines**
3.1. Timeliness of diagnosis
3.2. Timeliness of treatment
3.3. Convenience of care
3.4. Interpersonal communication

### Systematic literature review

The primary purpose of the systematic review was to give a general overview about the representation of PPVs in guidelines. The following keywords were identified based on the survey and the explotarory research: (malignan^*^[Title/Abstract] OR oncolog^*^[Title/Abstract] OR cancer[Title/Abstract]) AND (guideline[Title/Abstract]) AND (patient empowerment OR patient value OR patient perspective OR patient preference OR patient centered OR patient view). Pubmed was used as a search engine; search strategy was finalized on 2nd September 2016. Title and abstract screening was done by two independent reviewers by adapting the PRISMA checklist (Jahan et al., 2016). The PICOS—Population-, Intervention-, Comparison-, Outcome- and Study design-based—criteria, originally developed to randomized clinical trials, were restricted to intervention- and study design-based elements. All oncology guidelines focusing on cancer care-related interventions met our inclusion criteria. Hits were further restricted to English language materials published in the last 5 years designated as guidelines by the Pubmed engine. Guidelines eligible for full text review were subject of data extraction.

## Results

### Survey results

#### Characteristics of patient organizations

Respondents to the survey were representatives of 19 POs located in 18 countries (Argentina, Belgium, Brazil, Bulgaria, Canada (two POs), Croatia, Ireland, Italy, Japan, Lithuania, Norway, Philippines, Poland, Romania, Spain, United Kingdom, United States and Venezuela). For list of POs involved in the survey, please check Table A1. Typical form of funding for the operation and activities of POs was reported to be dominantly private. Budget constraints resulted in a relatively low number of paid employees (typically fewer than five persons per organization), so the majority of organizations had to rely on non-paid volunteers. The general characteristics of POs are described in Table 2.

**Table 2:** General characteristics of Patient Organizations that participated in the survey.

**Region**	**Country**	**Funding sources of PO activities**	**Staff**	
			**Number of paid workers**	**Number of volunteers**
**Europe**	Lithuania	Mainly private	Under 5 paid workers	10–100 volunteers
	Italy	Mainly private	Under 5 paid workers	10–100 volunteers
	Poland	Mainly private	Under 5 paid workers	Under 10 volunteers
	Spain	Mainly private	Under 5 paid workers	10–100 volunteers
	Ireland	Mainly private	Under 5 paid workers	Under 10 volunteers
	UK	Mainly private	5–10 paid workers	No volunteer
	Romania	Mainly private	5–10 paid workers	Above 100 volunteers
	Belgium	Mixed private and public	No paid worker	Under 10 volunteers
	Bulgaria	Mixed private and public	No paid worker	Nd
	Norway	Mixed private and public	Under 5 paid workers	Under 10 volunteers
	Croatia	Mixed private and public	10–30 paid workers	10–100 volunteers
**North America**	USA	Mainly public	10–30 paid workers	Above 100 volunteers
	Canada(a)	Mainly private	5–10 paid workers	Under 10 volunteers
	Canada(b)	Mainly private	10–30 paid workers	Above 100 volunteers
**South America**	Venezuela	Mainly private	Nd	Nd
	Argentina	Mainly private	5–10 paid workers	10–100 volunteers
	Brazil	Mainly private	10–30 paid workers	10–100 volunteers
**Asia**	Japan	Mainly private	Under 5 paid workers	Nd
	Philippines	Mainly private	5–10 paid workers	Above 100 volunteers

*Nd, no data was available*.

#### Value propositions for patients

The three most valuable propositions of oncology care (where 1 is most important and 5 is less important) by survey respondents were “extended life” (mean value: 1.16), “treatment-free remission” (mean value: 1.37) and “pain reduction” (mean value: 1.42) (see Table 3). “Radical end-stage treatment with adverse events” (mean value: 3.68) and “possibility to take therapy without food” (mean value: 3.74) were the least preferred value propositions.

**Table 3:** Aspects of care and their importance in the survey (survey results).

**When receiving cancer treatment therapy, what would be the most important for you to achieve?**	**Importance 1 very important, 5 less important Mean Value (*SD*)**
Extended life	1.16 (1.0)
Treatment-free remission	1.37 (0.90)
Pain reduction	1.42 (1.15)
Reducing nausea due to your treatment	1.89 (1.15)
Return to work	2 (1.57)
Reducing fatigue	2.11 (1.10)
Participating in family events and leisure activities	2.11 (0.43)
Taking oral therapy rather than an injection	2.47 (1.06)
Possibility to have dose dispensed once monthly	2.63 (0.68)
Stress management support	2.68 (1.12)
Ability to take therapy once a day (vs twice daily or more)	2.95 (1.18)
Using radical end-stage treatment (with adverse effects) if it can extend life by at least 2 months	3.68 (1.19)
Possibility to take therapy without food	3.74 (1.34)

The heterogeneity of cancer PPVs were reflected in the survey results. The variability was the highest related to “return to work” (*SD*: 1.57), “possibility to take therapy without food” (*SD*: 1.34) and “using radical end-stage treatment” (*SD*: 1.19). The relevance of these items seemed to be dependent on the personal preferences of individual patients. The most consistently judged values characterized by the lowest *SD* were “participating in family events and leisure activities” (*SD*: 0.43), and “possibility to have dose dispensed once monthly” (*SD*: 0.68).

When PO representatives were asked about the desired improvement of cancer care in the survey, side-effect management and quality of life related aspects were the most frequently mentioned categories (see Table 4). More efficient use of biomarkers to determine the adequate treatment was also underlined to achieve the appropriate treatment for the right patient at the right time, leading to better disease control and longer survival.

**Table 4:** Fields for improvement in cancer care (survey results).

**What do you think could be improved in the current (or the last) treatment you (or your loved one) have received in order for you (or your loved one) to live a quality life?**	**Frequency of mentioning by POs**
Side-effect management	10
Better quality of life	10
Efficient use of biomarkers	5
Treatment-free remission	4
Less costly treatment	3
Emotional support	3
Less toxic treatment	2
Treatment tailored to the needs of individuals	2
Research on drug interactions	1
Oral therapy rather than injection	1
Less placebo treatment	1

#### Patient inclusion at macro-level decision-making

According to respondents, one of the most relevant areas where patient engagement should be more intense is HTA. Nevertheless, five of the 19 PO respondents reported limited use of HTA for policy and reimbursement decisions in their country (see Table 5). In some countries patient empowerment in HTA or policy recommendations was only superficial. In three cases POs did not contribute to final HTA recommendations or reimbursement decisions; their role was limited to participation in quality of life surveys. Three respondents who personally participated in HTA or policy discussions reported doubts whether their opinion was taken into account in the final decision.

**Table 5:** Involvement of patient representatives to health technology assessment (HTA) (survey results).

**Could you please list any examples from your country where patients and/or caregivers were involved in a health technology assessment (HTA) of cancer medicines, or other medications that cancer patients may need?**	**Frequency of mentioning by POs**
Participation at reimbursement committee discussion or HTA meetings	9
Limited use of HTA in the country	5
Involvement of patients to surveys in the HTA process	3
Patients can submit requests or dossiers for HTA committees or agencies	3
Voting right to HTA recommendation or reimbursement decisions	1
Patients are not involved or represented in the HTA process	2
Patients are involved into the HTA process, but it is uncertain whether their opinion is taken into account in the final recommendation	3

### Systematic review of clinical cancer care guidelines

#### Overview

**Figure 1:**
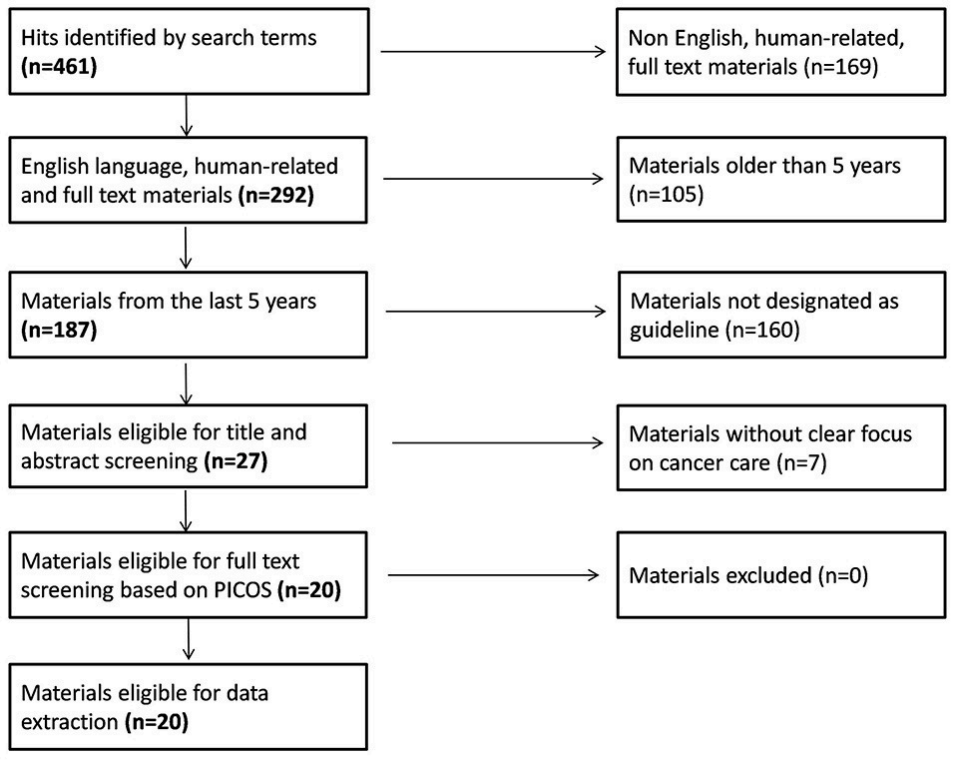
Flowchart of the systematic review.

Initially 461 articles were identified on Pubmed by our search terms. After restricting the hits to English language guidelines published in the last 5 years, 27 articles were eligible for title/abstract screening. By applying our pre-specified inclusion and exclusion criteria, 20 guidelines remained for full-text review and extraction of content (National Institute for Health and Care Excellence, 2011; Hurkmans et al., 2012; Moss et al., 2012; Watanabe et al., 2012; Carter et al., 2013; Qaseem et al., 2013; Thompson et al., 2013; Andersen et al., 2014; Freedland et al., 2014; Fukukita et al., 2014; Levy et al., 2014; Partridge et al., 2014; Wolff et al., 2014; Lebbe et al., 2015; Min et al., 2015; Steele et al., 2015; Stratigos et al., 2015; Tot et al., 2015; Young et al., 2015; Harris et al., 2016) (see Figure 1 for the full flowchart and Table 6 for study characteristics). The fields of breast, prostate and colorectal cancers dominated the therapeutic areas, while only four publications dealt with cancer in general terms. Half of the guidelines were addressing treatments, some described processes for screening and only a few guidelines covered both screening and treatment.

**Table 6:** Reviewed guidelines.

**Publication**	**Association**	**Country perspective**	**Short description**	**Therapeutic area**
Harris et al., 2016	ASCO	USA	Guideline for the use of breast tumor biomarker assay to choose the appropriate adjuvant therapy for women with early-stage invasive breast cancer	Breast cancer
Steele et al., 2015	ASCRS	USA	Practice guideline for the surveillance of patients after curative treatment of Colon and rectal cancer	Colorectal cancer
Freedland et al., 2014	ASCO	USA	Clinical practice guideline on adjuvant and salvage radiotherapy after prostatectomy	Prostate cancer
Levy et al., 2014	NCCN	Worldwide level	NCCN guidelines provide interdisciplinary recommendations on palliative care for patients with cancer.	Palliative care
Partridge et al., 2014	ASCO	USA	Practice guideline on chemo- and targeted therapy for women with HER2 negative (or unknown) advanced breast cancer	Breast cancer
Andersen et al., 2014	ASCO	Canada	Pan-Canadian practice guideline on screening, assessment and care of psychosocial distress	Psychosocial distress
Thompson et al., 2013	AUA/ASTRO	USA	Guideline on the use of radiotherapy after radical prostatectomy	Prostate cancer
Carter et al., 2013	AUA	USA	Guideline providing recommendations to urologists for the early detection of prostate cancer	Prostate cancer
Qaseem et al., 2013	CGC of ACP	USA	Guidance statement on prostate screening	Prostate cancer
National Institute for Health and Care Excellence (2011)	NCC-C	UK	Clinical guideline on the diagnosis and management of colorectal cancer	Colorectal cancer
Hurkmans et al., 2012	NVRO	Netherlands	Guideline for the radiotherapy of oncology patients with a pacemaker or ICD (Implantable Cardioverter Defibrillator)	Cancer patients with pacemaker or ICD
Fukukita et al., 2014	JSNM	Japan	Criteria for the data acquisition protocol for oncology (FDG-Pet/Ct) scans with the purpose of standardization	FDG-PET scans
Min et al., 2015	NCCSGDC	Korea	Korean guideline on cervical cancer screening	Cervical cancer
Moss et al., 2012	IARC	EU	Guidelines on colorectal cancer screening	Colorectal cancer
Wolff et al., 2014	ASCO	USA	Guideline for HER2 testing in the field of breast cancer	Breast cancer
Young et al., 2015	PCRWG	USA	Prostate cancer referral guideline	Prostate cancer
Tot et al., 2015	EBC	EU	Guideline setting up recommendations in the field of breast cancer pathology	Breast cancer
Watanabe et al., 2012	JSCCR	Japan	Guidelines for the treatment of colorectal cancer	Colorectal cancer
Lebbe et al., 2015	EDF + EADO	EU	Guideline on Merkel Cell Carcinoma diagnosis and management	Skin cancer
Stratigos et al., 2015	EDF + EADO	EU	Guideline on Diagnosis and treatment of invasive squamous cell carcinoma	Skin cancer

#### Patient empowerment related PPVs in the guidelines

**Figure 2:**
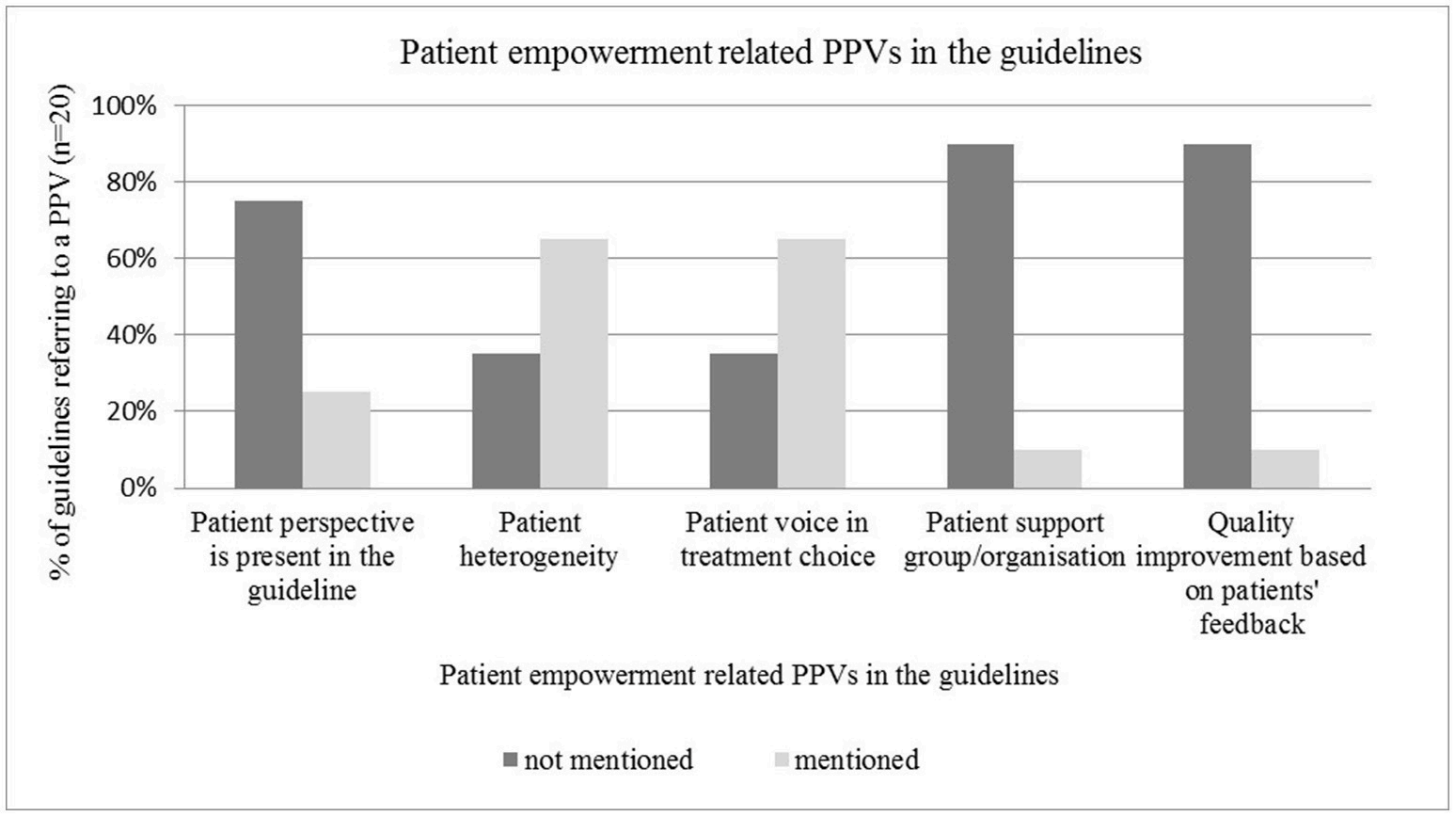
Patient empowerment related PPVs in the guidelines.

Patient involvement in treatment choice and recognition of patient heterogeneity were frequently addressed in the guidelines, such as by Freedland et al. (2014), Levy et al. (2014), Steele et al. (2015), and Harris et al. (2016). PPVs were represented in micro-level type decisions, such as involvement of individual patients in treatment choices (*n* = 13), rather than in macro-level type decisions, such as guideline development (*n* = 5). Active involvement of patients or patient representatives to guideline development was mentioned in only three publications (focus group, membership in the expert panel, interview with cancer patients). Two other guidelines only implicitly referred to the importance of the patient perspective. Engagement of POs and quality improvement based on PPVs seemed to be neglected topics in guidelines (*n* = 2 and *n* = 2 respectively) (see Figure 2).

#### Health outcomes related PPVs in the guidelines

**Figure 3:**
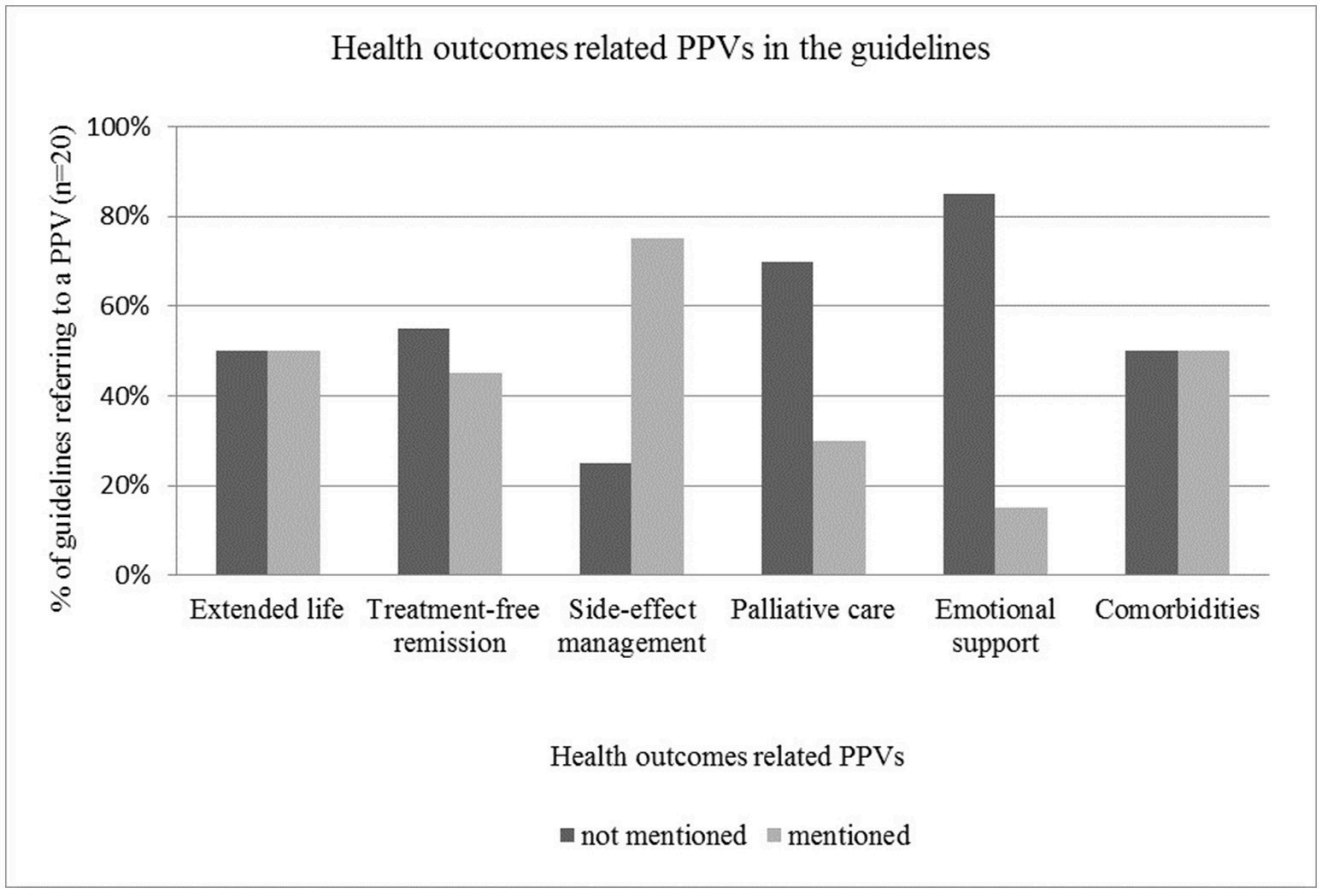
Health outcomes related PPVs in the guidelines.

Therapeutic management of side-effects was the most frequently included aspect of health outcomes in the guidelines with a mentioning frequency of 15, while aspects that were not strongly related to clinical treatment, such as palliative care and emotional support were rarely mentioned in the guidelines (*n* = 6 and *n* = 3 respectively) (see Figure 3).

The majority of the reviewed guidelines—such as by Hurkmans et al. (2012), Moss et al. (2012), Watanabe et al. (2012), Qaseem et al. (2013), Min et al. (2015), Young et al. (2015), and (National Institute for Health and Care Excellence, 2011)—highlighted that physicians must take into account the risk profile of therapeutic options (e.g., possible complications, adverse events and serious side-effects) and must inform the patient about all associated risks. However, detailed guidance for side-effect management was not usually provided. Half of the reviewed guidelines—including those by Hurkmans et al. (2012), Watanabe et al. (2012), Qaseem et al. (2013), Thompson et al. (2013), Partridge et al. (2014), Stratigos et al. (2015) and other publications—provided recommendations related to comorbidities. This aspect was addressed mainly from the perspective of how comorbidities influence treatment pathways or how they change the efficacy and benefit of primary therapies. Potential interactions between primary treatment and therapeutic management of comorbidities were also mentioned. Treatment-free remission and extended life were mentioned as patient values roughly in 50% of the cases (*n* = 9 and *n* = 10 respectively).

#### Patient management related PPVs in the guidelines

**Figure 4:**
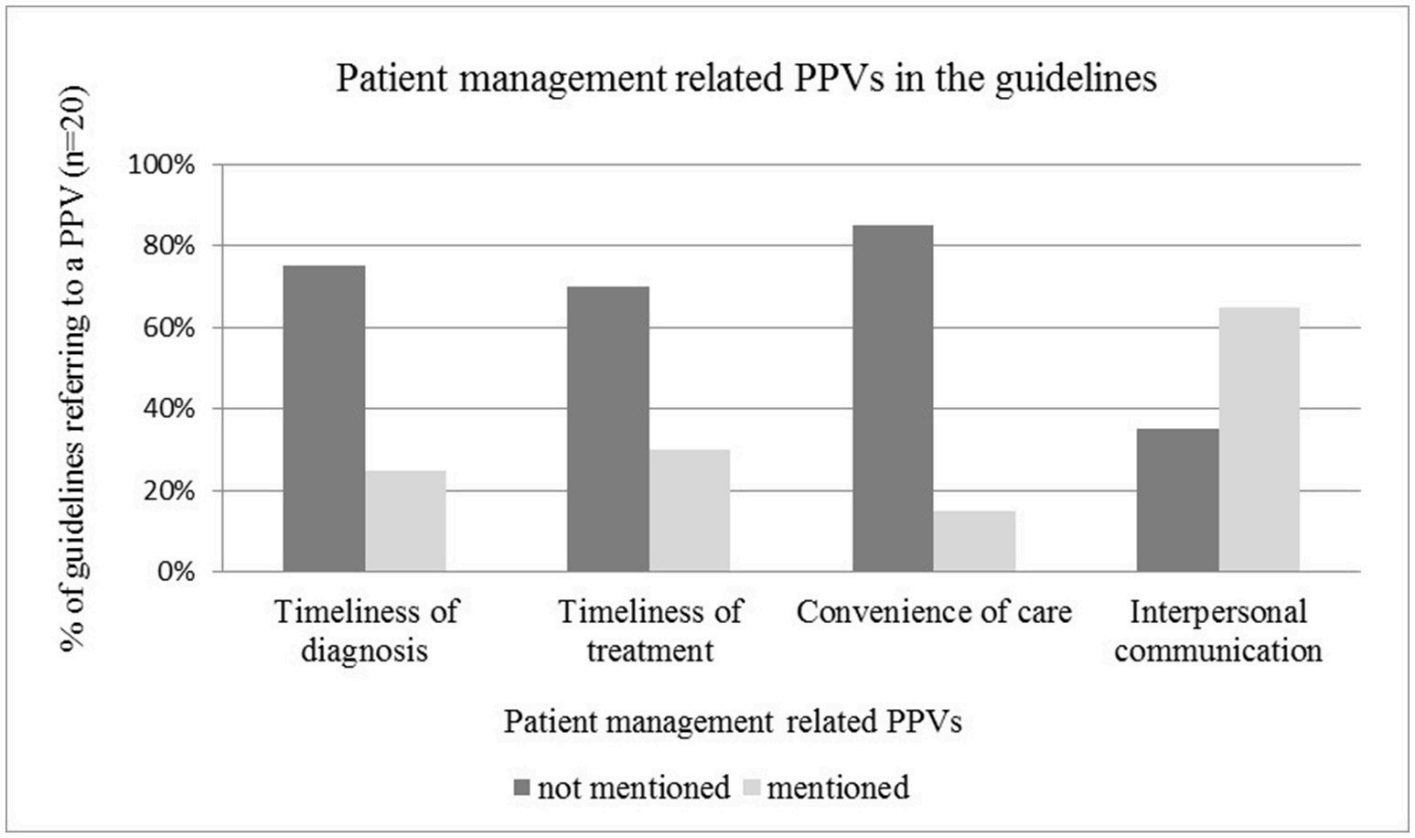
Patient management related PPVs in the guidelines.

The time window between initial and final diagnosis, and timeliness of treatment initiation as an individual level patient value was mentioned only in one quarter of the guidelines reviewed, highlighting the need for more explicit guidance on shortening waiting times for patients (see Figure 4). The relevance of interpersonal communication as patient value between individual patients and health care professionals was acknowledged in the majority of guidelines, such as in those by Moss et al. (2012), Carter et al. (2013), Andersen et al. (2014), Levy et al. (2014), Partridge et al. (2014) (see Figure 4). Reduced information asymmetry between patients and physicians by using easy-to-understand language and providing a detailed information package was highlighted in many guidelines. In some cases the emotional aspects of communication (*n* = 3) were also addressed PPVs, such as empathy, or referral to psychologists or support groups. The convenience of care was rarely mentioned as a PPV(*n* = 3).

## Discussion

### Patient involvement in guideline development and HTA decisions

Survey results indicated that the existence of a public HTA agency correlates with patient involvement with an exception of two countries. According to the systematic review, in cases where the process of patient engagement in guideline development was explicitly described, micro level PPVs were represented more significantly. This underscores the importance of including the patient voice in the discussion of a vast number of issues related to cancer care. To ensure effective input from patients and respective POs, both patients and the POs need to be better educated and informed to effectively engage in the evaluation or assessment process. Various ongoing initiatives, such as the European Patients Academy on Therapeutic Innovation (EUPATI) organize seminars to educate patients to participate in scientific and policy discussions and advocacy. Participation of patients and POs must be supported by easy-to use tools that consider the individual perspective of the patient in the development phase of clinical guidelines and policy recommendations. The instances of patient inclusion either at the institutional level (e.g., through patient involvement in the activities of the European Medicines Agency (EMA) in the European Union, the more fragmented approach by the Food and Drug Administration (FDA) in the United States, or the bottom-up approach taken by the National Institute for Health and Care Excellence (NICE) in the United Kingdom) or via *ad-hoc* participatory models attempted at the national level in certain countries, often lack meaningful participation and expectations from all stakeholders. Among ASCO (Schnipper et al., 2015, 2016), ICER (2017), MSKCC (2017), and NCCN (2017), only ICER included patients directly in the developmental process (Milken Institute, 2017). These value frameworks are the catalysts for addressing evaluation of expensive cancer treatments; however, much work needs to be done for those efforts to provide value to the actual patients, such as: (I) increase the overall document clarity by providing a secondary release of the same documents written in patient-friendly language, presented in a clear manner, (II) include PPVs, which may require inclusion of items outside the clinical realm, (III) meaningfully include the burden of illness on households and (IV) patient heterogeneity in value consideration.

### Patient heterogeneity

Frequent referencing of patient heterogeneity in the guidelines correlates well with the survey results; patients have various levels of disease burden, cancer stages, individual values and cultural backgrounds that equally affect their personal choices. There is also a strong consensus that the treatment strategy for each patient has to be determined jointly by the physicians and the patient on micro level, as needs and preferences of individual patients can be different (Elwyn et al., 2012; Martinez et al., 2015). However—contrary to the guidelines—survey findings showed that personalized medicine was not yet perceived as a crucial area of cancer care among survey respondents, which seems to be contradictory to recent trends in cancer care (CMBRP, 2016).

### Quality improvement in cancer care

Based on our survey results, feedback from patients was rarely taken into consideration while aiming at improving quality of cancer care, even though such survey tools had already been developed in the oncology field (Booij et al., 2013; Tremblay et al., 2015; Windham et al., 2015; King et al., 2016). This supports the contention that while patient-centricity is enshrined in global (via World Health Organization), regional (e.g., European Union) and national healthcare policies, it has yet to take shape in practice. In comparison to health care, in the field of consumer products, although fueled by commercial interests, the practice of seeking direct feedback from consumers on what is relevant, appealing, and satisfying to them has been globally established and a recognized model of action for decades. However, such practices are not generally prevalent in the cancer field, despite the fact that the impact of decisions made by health policy makers, experts at HTA level or healthcare professionals directly impact the lives of patients and their families. Quality in cancer care may be improved by further utilization of PREMs. Patient satisfaction measured by PREMs may be an important criterion in performance based reimbursement of health care providers based on current discussion in several countries, including the United States (Weldring and Smith, 2013) and the United Kingdom (Taylor et al., 2015; Burns et al., 2016).

### Patient preferences on health outcomes

Based on our survey results we cannot conclude that there is any aspect of cancer care referred to in the survey that is not relevant to the cancer patients. There were no items with >3.8 mean value, indicating the importance of each cancer care related item. Key value propositions (e.g., extended life, treatment-free remission and pain reduction) are in line with current value frameworks developed by ASCO, ESMO, ICER, MSKCC, and NCCN. Paradoxically, around only half of the reviewed guidelines addressed extended life, treatment-free remission and palliative care aspects of the treatment cycle. There are two potential explanations for this: either these objectives are too obvious for cancer care, or guidelines for cancer screening were not dealing with such requirements.

### Managing side effects

Survey findings highlighted the necessity of guidance for managing side effects, but clinical guidelines seemingly addressed this need. However, although serious adverse events and acute complications were mentioned, long-term and daily life affecting mild-to-moderate side-effects were usually lacking fields in the reviewed publications. This finding is in line with Basch et al. (Basch, 2016) stating that “chronic low-grade toxicities” are mostly ignored in current value frameworks. Nonetheless, initiatives to assign more importance to such mild-to moderate and daily life-affecting adverse events are on the horizon. ASCO—due to the high volume of comments received after having published their framework (Schnipper et al., 2015)—updated the original version and included also mild-to moderate adverse events in the updated framework.

### Timeliness

Interestingly, timeliness of diagnosis and treatment was rather underrepresented in current guidelines and neither was mentioned in the survey as field for improvement by PO representatives. However, previous survey results indicated the high relevance of these aspects of care according to Booij et al. (2013) who found that the average importance score of “early treatment initiation after diagnosis” and the item of “being referred to hospital as quickly as you would like” were the two most important quality aspects of care, being assigned equally 3.72 points on a 4-point Likert scale by cancer patients. If the diagnosis or treatment is delayed due to waiting lists, the patient may not be able to benefit from the treatment, and so scarce health care resources could have been better spent elsewhere (Rutqvist, 2006).

### Interpersonal communication

The relevance of interpersonal communication was mentioned in 13 guidelines. However, neither the guidelines nor the survey respondents highlighted the need for more efficient emotional support. This is in line with the findings of Booij et al. (2013) and Windham et al. (2015) who both identified psychological and emotional support as being underrated by survey respondents: Booij et al. found that the average importance score of the item entitled “It is regularly checked if you need help dealing with the emotions brought about by the disease and treatment” was only about 2.81 on a 4-point Likert scale. Windham et al. (2015) noted that the mean frequency rating of the item “Having your doctors be sensitive to your emotional reaction when telling you your diagnosis or test results” was only 4.54 on a 5-point Likert scale, being placed 20th on a list of 24 items.

## Limitations

The generalizability of our exploratory research is limited due to several factors. Search terms in the literature review were primarily focusing on guidelines providing direct therapeutic recommendations; thus the relatively low representation of patient aspects was not unexpected. This exploratory study was based on a limited number of survey respondents from 19 POs from 18 countries, where leaders of POs expressed their own views on the questions. Due to the limited number of included publications we have not assessed the abovementioned issues in different cancer types, therefore we could draw only general conclusions on the importance of patient engagement in cancer care.

## Conclusion

The PPVs in cancer care and treatment indicated by the surveyed POs were in line with current (HTA) practices, in which priority is given to overall and progression-free survival, reduced side-effects and improved quality of life. Patient involvement was mostly represented at micro-level decision-making processes, in the treatment planning phase, compared to the macro-level guideline development period. PPVs in cancer care guidelines were mostly limited to those micro-level aspects that are strictly related to health care provision (e.g., side-effects, comorbidities), or manifested in general terms. Although several validated PREMs exist, patient experience was a relatively neglected field and was mostly limited to emphasing the importance of interpersonal communication. Soft fields of patient aspects, such as emotional support, convenience of care were not considered thoroughly in the reviewed guidelines.

POs believe that patients should be encouraged to take an active role in their own care. Due to the heterogeneity of cancer patients and PPVs, developers of guidelines should engage patients and the respective POs systematically to ensure that PPVs are taken into account. Even if patient-centricity is a leading paradigm in cancer policy, it is not yet standard practice to include patients and / or POs at all appropriate levels of decision-making processes that are related to their health and well-being. Patient engagement and measurement of patient experience should be an integral part of cancer care decision-making. This complexity must be reflected throughout policy making, avoiding a population level “one-size-fits-all” solution. The situation poses a great need for in-depth collaborative solutions with a tangible inclusion of patients and / or the POs throughout the process.

## Author contributions

SN initiated the research and drafted the survey with KY; AI, and TA reviewed the survey. ZK finalized the survey. TA drafted the search query for the literature review. AI and ZK reviewed the search query, SN and KY finalized the search query. TA and AI conducted the literature review. BS, KM, and YM provided input for the survey. TA, AI, ZK, and SN interpreted the literautre review results. TA and AI drafted the manuscript. SN, KY, and ZK finalized the manuscript. BS, KM, and YM reviewed and commented the manuscript. All authors read and approved the revised version of the manuscript.

### Conflict of interest statement

Syreon Research Institute received research grant from Lithuanian Cancer Patient Coalition (POLA) to conduct the systematic review part of this study. The content of this paper, as well as the views and opinions expressed therein are those of the Authors and not the organizations that employ them. The authors declare that the research was conducted in the absence of any commercial or financial relationships that could be construed as a potential conflict of interest.

## Abbreviations

PO: Patient Organization
PPV: Patient preferences and values
PREM: Patient reported experience measure.

## Appendix

**Table A1:** Details of Patient Organizations that participated in the survey.

**Name of patient organization**	**Location**	**Name of respondent**	**Position of respondent**	**Contact details**
Europacolon	United Kingdom	Geoffrey Henning	Director of Policy	geoffrey@europacolon.com
Norwegian Melanoma Patient Association	Norway	Roald Nystad	Chairman	roald@nystad.net
National association for CML Patients Aid	Poland	Euzebiusz Jan Dziwinski	Board member	dziwinski.e@gmail.com
National coalition of patient organizations	Spain	Ainhoa Garcia	Member of steering committee	agarcigo22@hotmail.com
Irish Haemophilia Society	Ireland	Lyndsey Oconelly	Outreach co-ordinator	connolly.lyndsey@gmail.com
Europa UOMO	Belgium	Erik Briers	Board member	erikbriers@europa-uomo.org
Firefly Children with Cancer	Croatia	Ana Radunic	Project manager	ana@krijesnica.hr
Funcamama	Venezuela	Adriana Curiel	Member of steering committee	acurielavila@gmail.com
Romanian National Community of Young Cancer Survivors	Romania	Daniel Tomai	Member of steering committee	danitomai@yahoo.com
GIST and STS Alliance for Patients	Bulgaria	Yuliana Popova	President	youlianapopova@yahoo.com
Lithuanian Cancer Patient Coalition	Lithuania	Šarūnas Narbutas	President	sarunas.narbutas@pola.lt
Ovarian Cancer Canada	Canada	Elisabeth Baugh	Chief Executive Officer	ebaugh@ovariancanada.org
Instituto Oncoguia	Brazil	Luciana Holz	President	lucianaholtz@oncoguia.org.br
Fundacion ACIAPO	Argentina	Ignacio Zervino	Board member	nacho nzervino@yahoo.com
PAN Can Japan	Japan	Yoshi Majima	President	yoshiyukimajima@gmail.com
Colorectal Cancer Association of Canada	Canada	Barry Stein	President	barrys@colorectal-cancer.ca
Prevent Cancer Foundation	USA	Carolyn Aldige	President	carolyn.aldige@preventcancer.org
ICANSERVE Foundation	Philippines	Kara Magsanoc —Alikpala	Founding President	karamalikpala@gmail.com
Women Against Lung Cancer in Europe	Italy	Stefania Vallone	President	stefania.vallone@womenagainstlungcancer.eu

## References

[B1] Al-Rohil R. N. Tarasen A. J. Carlson J. A. Wang K. Johnson A. Yelensky R.. (2016). Evaluation of 122 advanced-stage cutaneous squamous cell carcinomas by comprehensive genomic profiling opens the door for new routes to targeted therapies. Cancer 122, 249–257. 10.1002/cncr.29738 26479420

[B2] Andersen B. L. DeRubeis R. J. Berman B. S. Gruman J. Champion V. L. Massie M. J.. (2014). Screening, assessment, and care of anxiety and depressive symptoms in adults with cancer: an American society of clinical oncology guideline adaptation. J. Clin. Oncol. 32, 1605–1619. 10.1200/JCO.2013.52.4611 24733793 PMC4090422

[B3] Basch E. (2016). Toward a patient-centered value framework in oncology. JAMA 315, 2073–2074. 10.1001/jama.2016.4637 27187297

[B4] Booij J. C. Zegers M. Evers P. M. Hendriks M. Delnoij D. M. Rademakers J. J. (2013). Improving cancer patient care: development of a generic cancer consumer quality index questionnaire for cancer patients. BMC Cancer 13:203. 10.1186/1471-2407-13-203 23617741 PMC3648393

[B5] Burns K. Harle A. S. Blackhall F. Fenemore J. Yorke J. (2016). 196TiP: PREM-LC: development and pilot testing of a patient reported experience measure in lung cancer. J. Thorac. Oncol. 11(Suppl. 4):S142. 10.1016/S1556-0864(16)30305-727198332

[B6] Cancer Moonshot Blue Ribbon Panel, (CMBRP) (2016). Blue Ribbon Panel Report. Available online at: https://www.cancer.gov/research/key-initiatives/moonshot-cancer-initiative/blue-ribbon-panel (Accessed January 23, 2017).

[B7] Carter H. B. Albertsen P. C. Barry M. J. Etzioni R. Freedland S. J. Greene K. L.. (2013). Early detection of prostate cancer: AUA Guideline. J. Urol. 190, 419–426. 10.1016/j.juro.2013.04.119 23659877 PMC4020420

[B8] Cherny N. I. Sullivan R. Dafni U. Kerst J. M. Sobrero A. Zielinski C.. (2015). A standardised, generic, validated approach to stratify the magnitude of clinical benefit that can be anticipated from anti-cancer therapies: the European Society for Medical Oncology Magnitude of Clinical Benefit Scale (ESMO-MCBS). Ann. Oncol. 26, 1547–1573. 10.1093/annonc/mdv249 26026162

[B9] Elwyn G. Frosch D. Thomson R. Joseph-Williams N. Lloyd A. Kinnersley P.. (2012). Shared decision making: a model for clinical practice. J. Gen. Intern. Med. 27, 1361–1367. 10.1007/s11606-012-2077-6 22618581 PMC3445676

[B10] Epstein R. M. Street R. L. (2007). Patient-centered communication in Cancer Care - Promoting Healing and Reducing Suffering. Bethesda, MD: National Cancer Institute Available online at: https://healthcaredelivery.cancer.gov/pcc/pcc_monograph.pdf?file=/pcc/communication/pcc_monograph.pdf (Accessed January 23, 2017).

[B11] Epstein R. M. Street R. L. Jr.. (2011). The values and value of patient-centered care. Ann. Fam. Med. 9, 100–103. 10.1370/afm.1239 21403134 PMC3056855

[B12] Freedland S. J. Rumble R. B. Finelli A. Chen R. C. Slovin S. Stein M. N.. (2014). Adjuvant and salvage radiotherapy after prostatectomy: American society of clinical oncology clinical practice guideline endorsement. J. Clin. Oncol. 32, 3892–3898. 10.1200/JCO.2014.58.8525 25366677

[B13] Fukukita H. Suzuki K. Matsumoto K. Terauchi T. Daisaki H. Ikari Y.. (2014). Japanese guideline for the oncology FDG-PET/CT data acquisition protocol: synopsis of Version 2.0. Ann. Nucl. Med. 28, 693–705. 10.1007/s12149-014-0849-2 24859759 PMC4332454

[B14] Halpern M. T. Urato M. P. Kent E. E. (2016). The health care experience of patients with cancer during the last year of life: analysis of the SEER-CAHPS data set. Cancer 123, 336–344. 10.1002/cncr.30319 27654842

[B15] Harris L. N. Ismaila N. McShane L. M. Andre F. Collyar D. E. Gonzalez-Angulo A. M.. (2016). Use of biomarkers to guide decisions on adjuvant systemic therapy for women with early-stage invasive breast cancer: American society of clinical oncology clinical practice guideline. J. Clin. Oncol. 34, 1134–1150. 10.1200/JCO.2015.65.2289 26858339 PMC4933134

[B16] Hurkmans C. W. Knegjens J. L. Oei B. S. Maas A. J. Uiterwaal G. J. van der Borden A. J.. (2012). Management of radiation oncology patients with a pacemaker or ICD: a new comprehensive practical guideline in The Netherlands. Radiat. Oncol. 7:198. 10.1186/1748-717X-7-198 23176563 PMC3528416

[B17] Institute for Clinical Economic Review, (ICER) (2017). Value Assessment Framework. Available online at: http://icer-review.org/methodology/icers-methods/icer-value-assessment-framework (Accessed January 23, 2017).

[B18] IOM (2001). Institute of Medicine US. Crossing the Quality Chasm: A New Health System for the 21st Century. Washington DC: 2001 by the National Academy of Sciences.

[B19] Jahan N. Naveed S. Zeshan M. Tahir M. A. (2016). How to conduct a systematic review: a narrative literature review. Cureus 8:e864. 10.7759/cureus.864 27924252 PMC5137994

[B20] Jonsson B. Hofmarcher T. Lindgren P. Wilking N. (2016). The cost and burden of cancer in the European Union 1995–2014. Eur. J. Cancer 66, 162–170. 10.1016/j.ejca.2016.06.022 27589247

[B21] Kassebaum N. J. Arora M. Barber R. M. Bhutta Z. A. Brown J. Carter A. (2016). Global, regional, and national disability-adjusted life-years (DALYs) for 315 diseases and injuries and healthy life expectancy (HALE), 1990–2015: a systematic analysis for the Global Burden of Disease Study 2015. Lancet 388, 1603–1658. 10.1016/S0140-6736(16)31460-X 27733283 PMC5388857

[B22] Kimman M. Jan S. Yip C. H. Thabrany H. Peters S. A. Bhoo-Pathy N.. (2015). Catastrophic health expenditure and 12-month mortality associated with cancer in Southeast Asia: results from a longitudinal study in eight countries. BMC Med. 13:190. 10.1186/s12916-015-0433-1 26282128 PMC4539728

[B23] King S. Exley J. Parks S. Ball S. Bienkowska-Gibbs T. MacLure C.. (2016). The use and impact of quality of life assessment tools in clinical care settings for cancer patients, with a particular emphasis on brain cancer: insights from a systematic review and stakeholder consultations. Qual. Life Res. 25, 2245–2256. 10.1007/s11136-016-1278-6 27039304 PMC4980409

[B24] Lebbe C. Becker J. C. Grob J. J. Malvehy J. Del Marmol V. Pehamberger H.. (2015). Diagnosis and treatment of Merkel Cell Carcinoma. European consensus-based interdisciplinary guideline. Eur. J. Cancer 51, 2396–2403. 10.1016/j.ejca.2015.06.131 26257075

[B25] Levy M. H. Smith T. Alvarez-Perez A. Back A. Baker J. N. Block S.. (2014). Palliative care, Version 1.2014. Featured updates to the NCCN Guidelines. J. Natl. Compr. Cancer Network 12, 1379–1388. 10.6004/jnccn.2014.0136 25313178

[B26] Luengo-Fernandez R. Leal J. Gray A. Sullivan R. (2013). Economic burden of cancer across the European Union: a population-based cost analysis. Lancet Oncol. 14, 1165–1174. 10.1016/S1470-2045(13)70442-X 24131614

[B27] Martinez K. A. Kurian A. W. Hawley S. T. Jagsi R. (2015). How can we best respect patient autonomy in breast cancer treatment decisions? Breast Cancer Manage. 4, 53–64. 10.2217/bmt.14.47 25733982 PMC4342843

[B28] McGuire S. (2016). World Cancer Report 2014. Geneva, Switzerland: World Health Organization, International Agency for Research on Cancer, WHO Press, 2015. Adv. Nutr. 7, 418–419. 10.3945/an.116.012211 26980827 PMC4785485

[B29] Memorial Sloan Kettering Cancer Center (MSKCC). (2017). Drug Abacus Tool. Available online at: http://www.drugabacus.org (Accessed January 23, 2017).

[B30] Milken Institute (2017). Integrating the Patient Perspective into the Development of Value Frameworks. Available online at: http://www.fastercures.org/assets/Uploads/value-coverage-framework-March-2016.pdf (Accessed January 23, 2017).

[B31] Min K. J. Lee Y. J. Suh M. Yoo C. W. Lim M. C. Choi J.. (2015). The Korean guideline for cervical cancer screening. J. Gynecol. Oncol. 26, 232–239. 10.3802/jgo.2015.26.3.232 26197860 PMC4510341

[B32] Moss S. Ancelle-Park R. Brenner H. (2012). European guidelines for quality assurance in colorectal cancer screening and diagnosis. First Edition–Evaluation and interpretation of screening outcomes. Endoscopy 44(Suppl 3):SE49–SE64. 10.1055/s-0032-1309788 23012122

[B33] National Institute for Health and Care Excellence, (NICE) (2011). Colorectal Cancer: The Diagnosis and Management of Colorectal Cancer. Cardiff, NSW: National Collaborating Centre, 2011.

[B34] National Comprehensive Cancer Network, (NCCN) (2017). NCCN Clinical Practice Guidelines in Oncology (NCCN Guidelines) with NCCN Evidence blocks. Available online at: https://www.nccn.org/evidenceblocks (Accessed January 23, 2017).

[B35] Partridge A. H. Rumble R. B. Carey L. A. Come S. E. Davidson N. E. Di Leo A.. (2014). Chemotherapy and targeted therapy for women with human epidermal growth factor receptor 2-negative (or unknown) advanced breast cancer: American society of clinical oncology clinical practice guideline. J. Clin. Oncol. 32, 3307–3329. 10.1200/JCO.2014.56.7479 25185096 PMC6076042

[B36] Pitts P. J. Goldberg R. (2015). Undermining patient values: The ASCO value in cancer care task force framework. J. Commer. Biotechnol. 21, 10–14. 10.5912/jcb723

[B37] Qaseem A. Barry M. J. Denberg T. D. Owens D. K. Shekelle P. (2013). Screening for prostate cancer: a guidance statement from the clinical guidelines committee of the American college of physicians. Ann. Intern. Med. 158, 761–769. 10.7326/0003-4819-158-10-201305210-00633 23567643

[B38] Rutqvist L. E. (2006). Waiting times for cancer patients–a slippery slope in oncology. Acta Oncol. 45, 121–123. 10.1080/02841860600549204 16546856

[B39] Schnipper L. E. Davidson N. E. Wollins D. S. Blayney D. W. Dicker A. P. Ganz P. A.. (2016). Updating the American Society of Clinical Oncology value framework: revisions and reflections in response to comments received. J. Clin. Oncol. 34, 2925–2934. 10.1200/JCO.2016.68.2518 27247218

[B40] Schnipper L. E. Davidson N. E. Wollins D. S. Tyne C. Blayney D. W. Blum D.. (2015). American Society of Clinical Oncology statement: a conceptual framework to assess the value of cancer treatment options. J. Clin. Oncol. 33, 2563–2577. 10.1200/JCO.2015.61.6706 26101248 PMC5015427

[B41] Siegel R. L. Miller K. D. Jemal A. (2015). Cancer statistics, 2015. Cancer J. Clin. 65, 5–29. 10.3322/caac.21254 25559415

[B42] Steele S. R. Chang G. J. Hendren S. Weiser M. Irani J. Buie W. D.. (2015). Practice guideline for the surveillance of patients after curative treatment of colon and rectal cancer. Dis. Colon Rectum. 58, 713–725. 10.1097/DCR.0000000000000410 26163950

[B43] Stratigos A. Garbe C. Lebbe C. Malvehy J. del Marmol V. Pehamberger H.. (2015). Diagnosis and treatment of invasive squamous cell carcinoma of the skin: European consensus-based interdisciplinary guideline. Eur. J. Cancer 51, 1989–2007. 10.1016/j.ejca.2015.06.110 26219687

[B44] Taylor R. M. Fern L. A. Solanki A. Hooker L. Carluccio A. Pye J.. (2015). Development and validation of the BRIGHTLIGHT Survey, a patient-reported experience measure for young people with cancer. Health Qual. Life Outcomes. 13:107. 10.1186/s12955-015-0312-7 26216214 PMC4517652

[B45] Thompson I. M. Valicenti R. K. Albertsen P. Davis B. J. Goldenberg S. L. Hahn C.. (2013). Adjuvant and salvage radiotherapy after prostatectomy: AUA/ASTRO guideline. J. Urol. 190, 441–449. 10.1016/j.juro.2013.05.032 23707439

[B46] Tot T. Viale G. Rutgers E. Bergsten-Nordstrom E. Costa A. (2015). Optimal breast cancer pathology manifesto. Eur. J. Cancer 51, 2285–2288. 10.1016/j.ejca.2015.06.127 26283037

[B47] Tremblay D. Roberge D. Berbiche D. (2015). Determinants of patient-reported experience of cancer services responsiveness. BMC Health Serv. Res. 15:425. 10.1186/s12913-015-1104-9 26416612 PMC4587918

[B48] van Baal P. H. Polder J. J. de Wit G. A. Hoogenveen R. T. Feenstra T. L. Boshuizen H. C.. (2008). Lifetime medical costs of obesity: prevention no cure for increasing health expenditure. PLoS Med. 5:e29. 10.1371/journal.pmed.0050029 18254654 PMC2225430

[B49] Vineis P. Wild C. P. (2014). Global cancer patterns: causes and prevention. Lancet 383, 549–557. 10.1016/S.0140-6736(13)62224-2 24351322

[B50] Wang H. Naghavi M. Allen C. Barber R. M. Bhutta Z. A. Carter A. (2016). Global, regional, and national life expectancy, all-cause mortality, and cause-specific mortality for 249 causes of death, 1980–2015: a systematic analysis for the Global Burden of Disease Study 2015. Lancet 388, 1459–1544. 10.1016/S0140-6736(16)31012-1 27733281 PMC5388903

[B51] Watanabe T. Itabashi M. Shimada Y. Tanaka S. Ito Y. Ajioka Y.. (2012). Japanese Society for Cancer of the Colon and Rectum (JSCCR) guidelines 2010 for the treatment of colorectal cancer. Int. J. Clin. Oncol. 17, 1–29. 10.1007/s10147-011-0315-2 22002491

[B52] Weldring T. Smith S. M. S. (2013). Patient-Reported Outcomes (PROs) and Patient-Reported Outcome Measures (PROMs). Health Serv. Insights 6, 61–68. 10.4137/HSI.S11093 25114561 PMC4089835

[B53] Windham A. Keller S. Yost K. Yang M. Mitchell D. Jenkins S. (2015). The Consumer-Based Cancer Care Value Index (CCCVI). Final Report, American Institutes for Research, Washington, DC.

[B54] Wolff A. C. Hammond M. E. Hicks D. G. Dowsett M. McShane L. M. Allison K. H.. (2014). Recommendations for human epidermal growth factor receptor 2 testing in breast cancer: American society of clinical oncology/college of American pathologists clinical practice guideline update. Arch. Pathol. Lab. Med. 138, 241–256. 10.5858/arpa.2013-0953-SA 24099077 PMC4086638

[B55] Young S. M. Bansal P. Vella E. T. Finelli A. Levitt C. Loblaw A. (2015). Guideline for referral of patients with suspected prostate cancer by family physicians and other primary care providers. Can. Fam. Physician. 61, 33–39. 25756141 PMC4301761

